# High-Sensitivity Whispering Gallery Mode Humidity Sensor Based on Glycerol Microdroplet Volumetric Expansion

**DOI:** 10.3390/s21051746

**Published:** 2021-03-03

**Authors:** Pauls Kristaps Reinis, Lase Milgrave, Kristians Draguns, Inga Brice, Janis Alnis, Aigars Atvars

**Affiliations:** Quantum Optics Laboratory, Institute of Atomic Physics and Spectroscopy, University of Latvia, LV-1586 Riga, Latvia; kristians.draguns@lu.lv (K.D.); inga.brice@lu.lv (I.B.); janis.alnis@lu.lv (J.A.); aigars.atvars@lu.lv (A.A.)

**Keywords:** whispering gallery mode, humidity sensor, microsphere resonator, glycerol, tunable laser

## Abstract

We demonstrate a highly sensitive whispering gallery mode (WGM) relative humidity (RH) sensor based on a glycerol microdroplet. WGMs were excited using a 760 nm tunable semiconductor laser. We used free space coupling, which is effective when using a liquid resonator. A detailed analysis of different parameters influencing the sensor’s characteristics (sensitivity, hysteresis, resolution, stability, and temperature) is presented. The sensitivity of the sensor is one of the highest reported (2.85 nm/% RH in the range 50–70% RH with the resolution 1 × 10^−4^% RH). This type of humidity sensor has several advantages, such as high sensitivity, extended lifetime, good repeatability, and low cost, as well as the use of a non-toxic and environmentally friendly liquid.

## 1. Introduction

Nowadays, precise humidity measurements play an important role in various sectors, such as industrial processing [1], the food industry [2], environmental control, automobile industry, the medical field [3,4], agriculture [5], and science [6]. However, commercially available electronic sensors are not always accurate or fast enough. Additionally, they become unstable in low and high relative humidity (RH) regions [7]. Whispering gallery mode (WGM) microresonators offer both fast measurement and high sensitivity in a larger range of humidity, as well as electromagnetic immunity [8]. The high sensitivity of WGM sensors is a result of high quality (*Q*) factors and low mode volumes [9]. *Q* factor largely depends on the application of different structures of resonators, such as microspheres, microbottles, microtoroids, microrings, and microdisks. All these cavities have smooth edges, they are transparent, and their refractive index must be higher than that of the surrounding environment [10].

When light inside the resonator reflects at an incident angle *θ* > *θ*_critical_, it gets trapped inside the cavity and, as a result, a continuous total internal reflection occurs [11]. Resonant photons travel inside the resonator and interact with the surrounding environment multiple times, thus increasing the sensitivity of the sensor [12]. Under these conditions, constructive interference takes place when only specific frequencies can survive, which leads to the resonance conditions for spherical microresonators, where the resonant wavelength *λ* depends on the radius *R* and the refractive index *n* of a spherical resonator [11]:

*λm* = 2π*Rn*, (1)

where *m* is the number of optical waves fitting into the circumference. These whispering gallery modes are named after an acoustic phenomenon discovered by Lord Rayleigh in the 19th century [13]. To achieve smaller radiative losses, cavities with a high refractive index should be chosen, as WGMs are trapped more effectively inside such cavities. There are many applications for WGM microresonators, such as spectroscopy and fluorescence studies, generation of frequency combs, biosensing, and many others [14].

**Figure 1:**
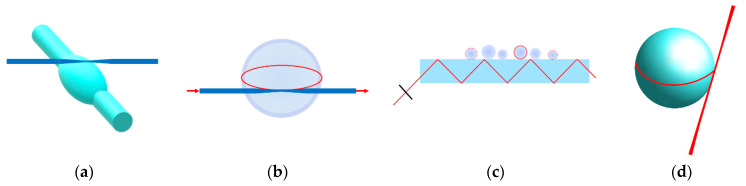
Different types of WGM humidity sensors: (**a**) Microbottle coupled by tapered fiber; (**b**) Microsphere coated with humidity-sensitive layer coupled by tapered fiber; (**c**) Many microspheres coupled by a prism; (**d**) Humidity sensitive liquid droplet resonator coupled by a free space beam.

Various studies report humidity sensing with microresonators (see Table 1 and Figure 1). Our paper explores a liquid glycerol resonator, which has higher sensitivity than the solid resonators [15]. Glycerol was chosen due to its transparency, viscosity, non-toxicity, and a low response to temperature fluctuations. For each RH, there are known equilibrium states of glycerol-water mixture [16]. RH represents the ratio of the partial pressure to the saturated water vapor at a specific temperature. Therefore, relative humidity is a temperature dependent variable and is expressed in percentages [17]. As glycerol is trivalent alcohol where 3 -OH groups are present, it can absorb moisture from the air. If material is sensitive to relative humidity, it is also sensitive to absolute humidity.

As glycerol is highly hygroscopic; it effectively absorbs water molecules as the relative humidity (RH) increases, which results in a wavelength shift due to the change in radius and refractive index:

Δ*λ*/*λ* = Δ*R*/*R* + Δ*n*/*n*. (2)

Because of this, it is possible to use a glycerol droplet as a high-sensitivity humidity sensor.

There are different advantages and disadvantages to all the sensors described in Table 1. The highest sensitivity is achieved with liquid resonators (this study confirms it) and the fabrication process is also easy. However, one should consider that because of the type of liquid, Brownian motion and surface capillarity phenomena could play a role [26]. Only one liquid resonator for humidity measurements has been reported so far, while different solid resonators have been proposed–both coated and uncoated. Coating in general increases the sensitivity but lowers the *Q* factor (on average by 1 order of magnitude). The thin coating can improve the sensor’s response time significantly. A study by Mehrabani et al. shows that thicker coating has a faster recovery time [24]. Resonators without coatings are easier to fabricate and can deliver good results as well, as shown by Petermann et al., who chose to use many polymethacrylate (PMMA) spheres to lower the necessity for high quality resonators, and the use of PMMA meant no coating was necessary [20]. Solid resonators differ in their shape—from spheres to complex nanomembranes—and all are created to find the best performance/ease of fabrication/price ratio.

We obtained the average sensitivity of 2.85 nm/% RH, which is the highest reported when compared to the results in Table 1. The closest is 2 nm/% RH, the only other liquid resonator by Labrador-Paez et al. [15]. Our achieved resolution is 1 × 10^−4^% RH, corresponding to 2 pm.

In this paper, a glycerol droplet as a temperature independent optical RH sensor with high sensitivity and long lifetime is demonstrated. A novel approach by using pure glycerol, tunable laser, and oscilloscope allows detecting changes in the surrounding environment as low as 1 × 10^−4^% RH. A detailed analysis of different parameters influencing the sensor’s characteristics (sensitivity, hysteresis, resolution, stability, and temperature) is presented. This type of humidity sensor has several advantages, such as high sensitivity, extended lifetime, good repeatability, and low cost, as well as the use of a non-toxic and environmentally friendly liquid. In particular, we demonstrate that the sensitivity achieved by this liquid resonator is several orders higher than that of solid resonators.

## 2. Materials and Methods

The method used for humidity detection is based on laser interference. We created a simple set-up that was easy to use. The main components of the set-up were a light source, a hygroscopic resonator, and a detector. The beam of the tunable VCSEL (vertical-cavity surface-emitting laser) 760 nm laser was fed into a single mode optical fiber (SMF) and transmitted into our custom-made climate chamber. The laser used was an AVAP 760 nm (SPECDILAS V-763-OXY, Laser Components GmbH, Olching, Germany) single-mode VCSEL, which provides exceptional tunability, low power, and low cost, thus making it promising for a portable and industrially applicable sensor (maximal output power 0.5 mW, 80 MHz linewidth, continuous tuning range by a linear current ramp ~200 GHz, sweep repetition frequency of 50 Hz). Based on the transmission spectra of glycerol [27] and water [28], 760 nm is an optimal choice. We experimentally measured the transmission spectra of glycerol, water, and 50:50 glycerol–water mix to confirm this. The obtained data can be viewed in the Supplementary Material, Figure S1. Additionally, the chosen wavelength does not cause photodegradation (as opposed to green light).

**Figure 2:**
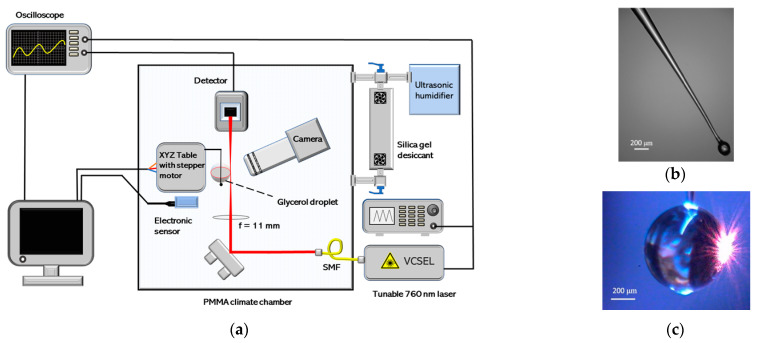
(**a**) Experimental set-up for humidity measurements. After entering the climate chamber, light is reflected from the mirror. The converging lens focuses the light on the side of the glycerol droplet. Photodiode collects WGM transmission spectrum and sends data to the oscilloscope that is operated in AC coupling mode. The electronic humidity sensor for control measurements is positioned a few centimeters away from the droplet; (**b**) Fine tapered fiber with a melted tip to support the droplet; (**c**) Glycerol microsphere created with dipping method. The laser beam was focused on the side of the glycerol droplet to excite the WGM resonances.

The climate chamber’s walls consisted of PMMA plates (impregnated with hydrophobic spray), which formed a box with dimensions: 44 cm × 44 cm × 39 cm. A commercially available ultrasonic humidifier and a home-made air desiccant container (filled with silica gel and powered by two fans) were connected to the PMMA chamber using tubes. A schematic view of the set-up is shown in Figure 2a. Humidity and temperature control measurements were made using TSP01 sensor (Thorlabs Inc., Newton, NJ, USA; precision ±2% RH (20% to 80% RH), resolution 0.1% RH, thermometer precision ±1 °C, resolution 0.05 °C).

Once the laser beam enters the climate chamber, it is focused on the side of the hygroscopic resonator using a converging lens (*f =* 11 mm). This free space coupling method has lower coupling efficiency than that of tapered fiber or prism, but it does not disturb the droplet [26]. Fused silica etalon (plate thickness *l =* 9.75 mm, *n =* 1.454) can be positioned in the laser beam instead of the resonator for calibration of laser frequency scanning.

The end of an optical fiber (SMF-28, Thorlabs) was melted and stretched to create tapered fiber with a slightly wider tip region to support the glycerol droplet (Figure 2b). Afterwards this fiber was dipped into the glycerol [15], which sticks to the prepared fiber (Figure 2c). Due to surface tension forces, a nearly perfect spherical droplet limited by Brownian motion was created [26,29]. We used this technique to have a stable support for the droplet, which is provided by having a wider upper diameter of the fiber. The melted tip is necessary due to the nature of the chosen material—the droplet moves to the widest part of the fiber—and by melting the tip, it is guaranteed to stay in a fixed position at the end of the fiber.

Experiments were conducted using a droplet with an equatorial radius of approximately 370 ± 15 μm at 50% RH. Equation (3), where *d*1 and *d*2 are the minor and major axes of the elliptic sphere, was used to evaluate the eccentricity [30]:

*e* = (*d*1 − *d*2)/(*d*1 + *d*2) × 100%, (3)

An eccentricity of 1.2% was calculated for the droplet shown in Figure 2c. At higher RH, droplets tend to have lower eccentricity, as they increase in volume and become more spherical.

The prepared fiber with the glycerol droplet on it was attached to the XYZ optical table. To control the movement of the droplet perpendicular to the laser beam, a stepper motor was used. This was necessary because the size of the droplet depends on RH. As glycerol absorbs water and grows bigger in size, the coupling position changes and the intensity of the signal decreases and finally the resonance disappears. At the same time, the signal intensity received by the photodiode decreases. The Python program was used to control the stepper motor and to adjust the position of the resonator, thus keeping the laser beam focused on the side of the resonator. We used a Thorlabs photodiode PDA36A with an active area of 13 mm^2^, which is large and therefore does not require an additional focusing lens to collect the transmission signal [31].

To create a WGM microresonator, glycerol (Riga Pharmaceutical Factory, Riga, Latvia) was chosen. It is a hygroscopic material with known glycerol-water proportions for each % RH [16]. It can be used as a humidity sensor due to its transparency, its insignificant dependency on temperature, and its selectivity. In addition, glycerol is non-volatile under normal atmospheric pressure [32]. Glycerol has several other advantages when compared to other hygroscopic materials (ethanol, concentrated sulfuric acid, etc.): it is non-toxic, non-irritating, odorless, highly viscose, and low priced. Glycerol, when placed in moist air, absorbs water and some gasses, such as hydrogen sulfide and sulfur dioxide [33], both present in specific environments, such as swamps and around volcanic activity. This makes glycerol highly selective when used for humidity measurements in more typical conditions and indoors.

To maintain constant RH for stability measurements and sensor calibration we used saturated salt solutions [34]. The use of NaCl solution stabilizes the humidity to 75% RH. We used common cooking salt and measured constant humidity of 72% RH using TSP01 sensor.

## 3. WGM Spectra Measurements

**Figure 3:**
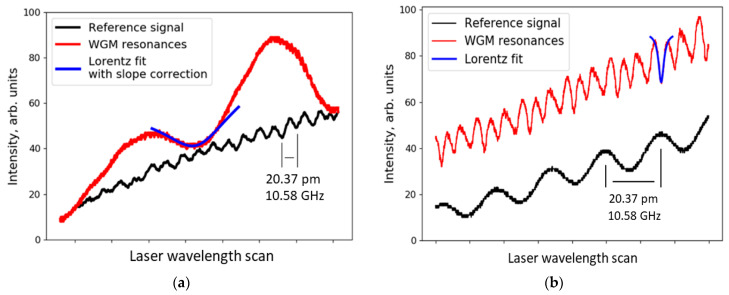
WGM resonance spectrum depends on the coupling conditions and the position of the focused light; (**a**) Fundamental modes with free spectral range (FSR) of 165 pm. Resonance depth was 10% of the total light intensity; (**b**) Spectrum of higher-order modes excited by focusing the light below the equator of the elliptic droplet.

The WGM spectrum excited by a 760 nm tunable laser is shown in Figure 3. A *Q* factor of 7.3 × 10^3^ was calculated for the fundamental modes. Deeper resonances with higher *Q* factors were observed at RH > 70% conditions. The coupling position also affects the resonances. By focusing light below the equator of the droplet, higher-order modes with *Q* factor up to 3.2 × 10^5^ were recorded, as shown in Figure 3b. Because these modes are more sensitive to alignment, we did not use them for RH measurements. Instead, fundamental modes as shown in Figure 3a were excited and used for RH detection. From the light absorption of 5%/cm in glycerol-water solution we calculated the 1/*e* decay length of 0.2 m. This gives the maximum absorption-limited quality factor *Q* = 0.2 m/760 nm = 3 × 10^5^ which agrees with our experimental observations.

**Figure 4:**
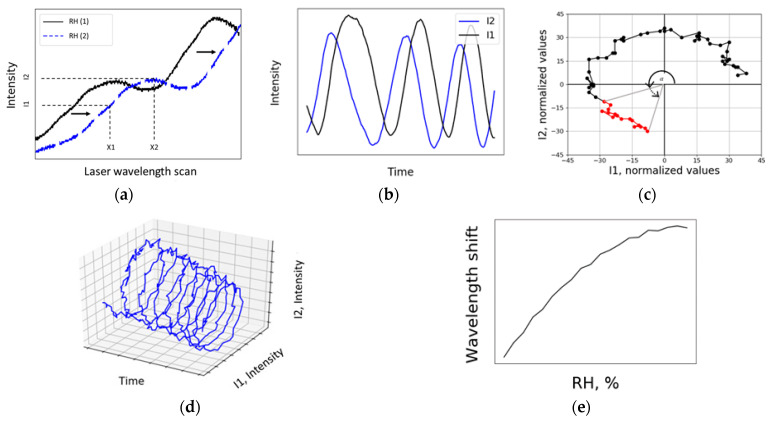
Illustrative principal steps of the data analysis method. The intensity was continuously recorded at two waveform points and Lissajous figure was plotted: (**a**) WGM resonance spectrum where the blue, dotted curve shows a shift to the right due to increase in RH. The black curve shows the resonance position at some initial RH. The intensity was recorded at points X1 and X2. Thus, I1 = f(X1) and I2 = f(X2); (**b**) As RH changed and modes shifted by, sinusoidal graphs were recorded. Sinusoidal functions reach their minimum when resonance peaks pass points X1 and X2. However, it is impossible to know if the humidity is increasing or decreasing from this graph; (**c**) Values I1 and I2 were normalized to create a circle (Lissajous figure) around the origin point (0, 0). By knowing X and Y projections (normalized I1 and I2 values), a radius vector could be constructed and an angle α could be determined. As RH increased, the angle α also increased and represented the shift in wavelength. The direction in which the normalized Lissajous figure is rotating determines whether RH is increasing or decreasing, while the modulus of angle α (which corresponds to wavelength shift) determines how much RH has changed; (**d**) 3D plot shows I1 and I2 in the time domain. Spiral-like structure was analyzed as a Lissajous figure; (**e**) Result of data processing—accumulated wavelength shift due to change in RH.

To analyze the above data (Figure 3a), the waveform from the oscilloscope was collected three times per second. The oscilloscope’s X-axis consists of 4000 data points. To determine if the humidity increased or decreased (modes shifted to the right or left accordingly), our developed method used the recorded intensity at points X1 and X2 (Figure 4a). Points were chosen so that one of them would be at a local minimum (analogous to sin(0)) and the other would have a phase difference of *π*/2. As a result, by plotting the values of X1 and X2 (values change in time as RH changes), two sinusoidal graphs were obtained (Figure 4b). To process the data, an original Python program was created. Using the two sinusoidal graphs, the Lissajous figure was plotted (Figure 4d) [35] and normalized based on the amount of light the detector received (Figure 4c). Without the normalized intensity values I1 and I2, the obtained Lissajous figure would form an impractical spiral. The angular shift in Lissajous figure represents the shift in resonant wavelength (Figure 4e). A shift of 360° equals a shift of one free spectral range (FSR). The developed method does not require very high *Q* factors for detecting the wavelength shift as it does not follow one peak. However, this method can be used efficiently only when the resonance half width at half maximum is similar to the half of FSR. Peaks should create smooth and sinusoidal-like curve.

The developed program can sustain the resonant signal without human interference. Stepper motor movements do not interfere with the program’s ability to measure the wavelength shift and thus the RH. This means that a new and fully automatic system, which can control the signal power, the position of the droplet, and process the data in real time, has been created.

The resonator’s stability in time and temperature change was analyzed. To understand if the resonator is stable in time, a 24-h test was conducted. Using NaCl saturated solution we maintained constant humidity of 72% RH and started the measurement. To experimentally demonstrate glycerol’s stability in temperature, we compared the data from two experiments: (1) RH was changed, no temperature change; (2) only temperature was changed in the chamber, leading to RH change (absolute humidity in this case is constant as the change happened in a closed chamber). These results are described in Section 4.

The experimental data was also compared with computer simulations. The simulations were made using the COMSOL Multiphysics Wave Optics Module with 2D axial-symmetrical mode (COMSOL Inc., Burlington, MA, USA). The model consists of a resonator with its radius and refractive index [36] being a function of relative humidity. The wavelength used for both the experiment and simulations was 760 nm. Around the resonator there is an area with a refractive index *n* = 1, to simulate the air surrounding the resonator.

To evaluate temperature influence on the glycerol droplet, a simulation was made at constant relative humidity 50% (R = 369 μm, *n* = 1.3981, volumetric thermal expansion coefficient α = 5 × 10^−4^ K^−1^ and thermo-optic coefficient β = –5 × 10^−4^ K^−1^), and temperature increased from 20 to 21 °C.

## 4. Results

We transformed the collected data from frequency shift to wavelength shift at 760 nm, where 1 pm = 0.52 GHz. Although the wavelength that we used stayed at 760 nm, we measured the wavelength shift by counting modes that crossed the oscilloscope screen.

### 4.1. Sensitivity and Resolution

**Figure 5:**
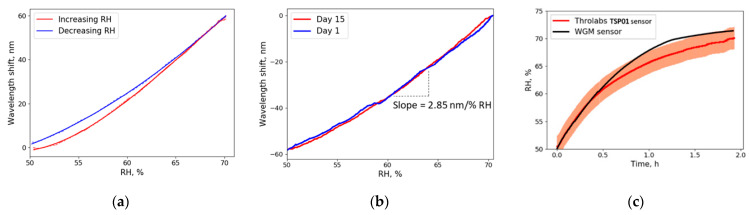
Recorded wavelength shift due to change in RH: (**a**) Humidity was slowly increased from 50% to 70% RH by using a saturated NaCl solution, which was placed inside the humidity chamber. Then NaCl was removed and humidity was reduced by circulating humid air through a container filled with silica gel desiccant. Continuous lines illustrate data fitted with the 3rd-order polynomial; (**b**) Wavelength shift recorded while reducing humidity on day 1 and day 15. The plot illustrates high repeatability and sensitivity (slope of the curves); (**c**) Comparison of TSP01 humidity sensor (Thorlabs) and proposed glycerol WGM sensor. The graph illustrates the measured RH values with both sensors. Humidity was slowly increased using saturated NaCl solution in the range 50–70% RH. The colored band represents the imprecision (±2% RH) of the Thorlabs sensor (see Section 2).

The wavelength shift shown in Figure 5 was mainly caused by the change in the droplet’s radius. Although the refractive index changes as well, its influence on the wavelength shift is approximately two times lower. As humidity increases, the wavelength shift becomes more rapid (radius changes exponentially, see Section 4.4) and the sensitivity of the resonator increases; therefore, the overall sensitivity is not linear. Sensitivity was obtained as the slope of the curves in Figure 5a,b. In the full range of RH (0–100% RH), the sensitivity is not linear, but close to linear when looking at smaller ranges of RH (for example, 50–60% RH). For a 50–70% RH change, approximately 350 resonance waves crossed the oscilloscope screen. The average sensitivity of 2.85 nm/% RH puts this sensor in front of other proposed optical RH sensors (see Table 1).

The precision of our sensor is limited by its response time (which depends on the volume of glycerol used. The thinner the layer around the fiber, the faster the response time), laser drift in time, data points received per second, and oscilloscope resolution. Assuming that experimentally obtained data creates a Lissajous figure with very low eccentricity, the precision of 7 × 10^−4^% RH was calculated by evaluating signal to noise ratio (SNR).

To evaluate the resolution limit for the proposed sensor, an equation demonstrated by Labrador-Paez et al. [15] was used:

Δ*RH_min_* = (Δ*λ_min_*)/(Δ*λ*/Δ*RH*). (4)

Experimentally, we measured VCSEL laser drift in time against molecular O_2_ line in the air. Without temperature stabilization, a drift of 160 MHz was detected. It was the main factor limiting the resolution and was used in Equation (4) as Δ*λ_min_*. Thus, the estimated resolution for the glycerol droplet sensor is 1 × 10^−4^% RH, which makes this sensor highly sensitive to RH changes in the surrounding environment. A larger droplet increases the sensitivity because for greater Δ*R* there will be greater Δ*λ*. However, larger droplets are more sensitive to vibrations and have slower response time.

### 4.2. Hysteresis, Repeatability and Stability

The obtained data showed low hysteresis (Figure 5a) and good repeatability (Figure 5b)—two of the most important parameters for sensing applications. In repeated experiments (15 days apart), the wavelength shift was stable, and the lines matched almost perfectly. The good hysteresis was due to the nature of water-glycerol equilibrium states at different humidity levels. Many other optical devices and solid resonators are extremely sensitive to dust particles [37,38]. Meanwhile, our sensor had a longer lifetime than demonstrated previously—experiments with the same microdroplet were conducted over a month-long period.

To get a better understanding of the glycerol sensor’s performance, we compared our sensor to the electronic sensor used. The curves of the Thorlabs sensor (TSP01) and the WGM sensor coincide within the margin of error (Figure 5c). In the 50–60% RH region, the WGM sensor and the Thorlabs sensor show almost identical results. In higher RH conditions the WGM sensor shows faster response time, but finally, both sensors converted to constant RH level.

### 4.3. Temperature Dependence

Many of the proposed optical sensors are sensitive to temperature [20,39] and thus cannot be used independently for precise RH determination. Glycerol is sensitive to relative humidity and has a very low thermal expansion coefficient [16]. The volumetric thermal expansion coefficient of pure glycerol is: *α =* 2.85 × 10^−4^ K^−1^, of water *α =* 2.06 × 10^−4^ K^−1^ [40]. Simulation was performed, and for a temperature change of 1 K (20–21 °C), the wavelength shift was two orders of magnitude lower than that of 1% RH change. Compared to PMMA microspheres [20,41], which are more often used as WGM sensors, the influence of the temperature on the glycerol droplet is much lower because of its high hygroscopicity.

**Figure 6:**
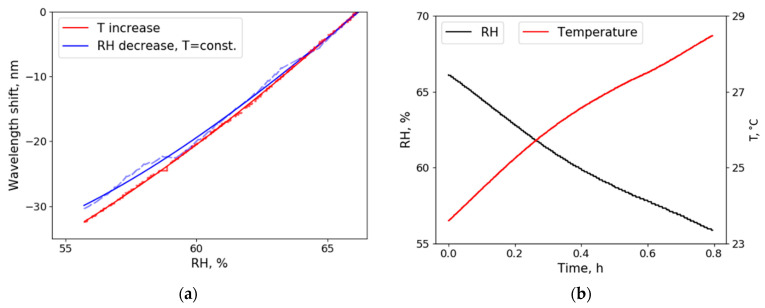
(**a**) Wavelength shift due to the RH change enforced only by dehumidifying the air (blue) and changing the temperature (red). Dotted lines illustrate raw data. To reduce RH, either silica gel desiccant or heating element (which increased the temperature from 23 to 28 °C) was used: The difference between the two curves represents the inaccuracy of the electric humidity sensor and the impact the temperature change has on the glycerol resonator. In an ideal case (no temperature dependence), both curves would match ideally; (**b**) Humidity and temperature change in the sealed chamber, when only temperature increased.

RH depends on both the amount of water vapor in the air and the temperature; therefore, RH can be controlled by manipulating one of these parameters. If the material used for sensing is temperature independent, wavelength shift (due to RH change) should be the same no matter how it is enforced. Figure 6a shows wavelength shift due to RH change enforced by reducing water vapor (blue curve) and by increasing temperature in the chamber (red curve). The difference between the two curves is caused partially due to the inaccuracy of the electric sensor used for control measurements and partially due to the small radius and refractive index changes created by temperature increase. Figure 6b shows the dependency of temperature and humidity in the climate chamber when the temperature was slowly increased (see Section 3; absolute humidity is constant, relative humidity changes due to the temperature change), and in this way, the red curve in Figure 6a was obtained. Such an experiment indicates that our proposed sensor is sensitive to relative humidity because absolute humidity was not changed while enforcing RH change only by temperature increase. When comparing Figure 6a,b, it can be seen that small temperature fluctuations by approximately 3 °C did not cause any additional wavelength shift (curves coincide), and larger temperature changes caused small wavelength shifts.

### 4.4. COMSOL Simulations

**Figure 7:**
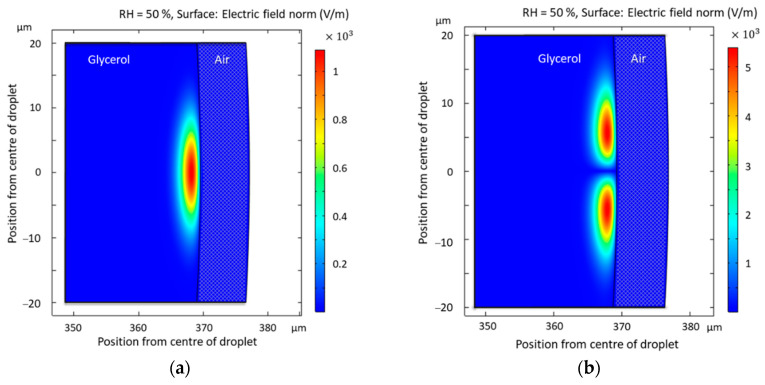
COMSOL simulations of whispering gallery modes in the glycerol droplet at 50% RH. Horizontal axis values represent the radius of the droplet. The dotted blue area on the right from the modes represents the surrounding environment (air): (**a**) Fundamental mode; (**b**) Non-fundamental mode.

COMSOL simulations of the excited modes in the glycerol droplet were made using a 2D axisymmetric model. We can see that the modes from the simulations (Figure 7) match with the experimentally recorded spectrum (see Figure 3a,b). It can be seen in Figure 7 that different order modes can be excited. This means that there are more peaks on the spectrum to track, thus increasing the accuracy of the sensor. We can also observe that our glycerol droplet has little to no evanescent field, meaning the radiation is contained within the resonator. This is a property of liquid resonators. The resonances are located near the surface and do not penetrate deeper than 5 μm into the glycerol droplet. To experimentally obtain higher-order modes, as shown in Figure 3b, the laser beam was focused slightly below the equator of the elliptic droplet.

**Figure 8:**
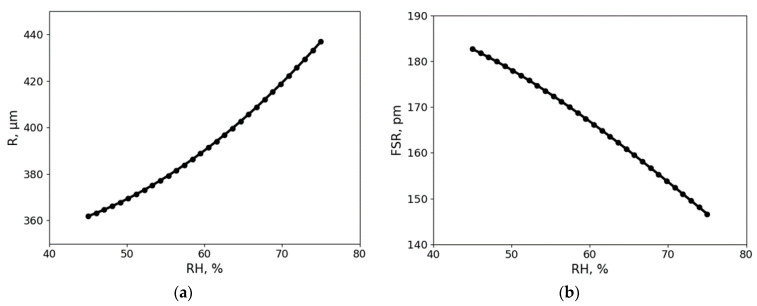
(**a**) The radius of the glycerol droplet at different RH levels. The curve illustrates the theoretically obtained values; (**b**) The resonator’s FSR at different RH levels also calculated theoretically.

As mentioned earlier, the change in radius of the glycerol droplet is the main parameter causing wavelength shift. When approaching higher RH levels, the radius increases exponentially. Thus, the sensitivity of the sensor also increases. In our experiments in the 45–75% RH range, the radius increased by 19% (Figure 8a). Glycerol solution has a known water/glycerol ratio for every RH level, which determines the refractive index. Additionally, each glycerol solution droplet at a specific RH has a unique radius. The volumetric expansion of this type of resonator is more significant than that of solid resonators with coating, making our resonator more sensitive. To make use of this property, the coupling position needed to be frequently adjusted. In future research, the measurement chamber can be made more compact and the coupling method can be adapted to create a sensor that is even easier to set-up and use. Tapered fiber can be used to induce resonances [42] and to keep the coupling position and the droplet stationed itself.

Due to change in both the refractive index and radius, relative humidity dependent FSR was observed experimentally. Theoretical values for FSR were calculated and are shown in Figure 8b. FSR decreases with the increase of RH. One of the benefits of the proposed glycerol droplet sensor is its ability to operate in humid conditions where the volumetric expansion of the glycerol droplet causes a significant shift in wavelength. Glycerol droplet sensor can also recover from saturated RH environment and keep its original properties. Combining these properties with the observed repeatability and stability over a 30-day period, a long lifetime sensor is created, which are necessary properties for any sensor and can be problematic in optical sensors.

## 5. Conclusions

In conclusion, we demonstrated that a liquid glycerol droplet can be used as a temperature-independent optical RH sensor with high sensitivity and long lifetime. The novel approach using pure glycerol, a tunable laser and an oscilloscope makes it possible to experimentally detect changes (resolution) in the surrounding environment as low as 7 × 10^−4^% RH with an average sensitivity of 2.85 nm/% RH. After calibration, one can follow the resonance shift in wavelength to determine RH. An original Python program for real-time data processing was created. Our method for constructing the Lissajous figure does not require high *Q* factors and reduces the impact of noisy data as the shift in wavelength is only detected if the intensity at both points X1 and X2 (see Figure 5a) changes according to the Lissajous figure equation, which means that noisy data only affect the length of the radius vector, and not the angle. A stepper motor controlled by a Python program automatically regulates the position of the glycerol droplet to sustain a continuous resonance signal. This program was able to endure a 24-h test and sustain a resonance signal as well as measuring the wavelength shift without interruptions. Such properties create opportunities for further research of glycerol droplet humidity sensors. This paper demonstrates that the sensitivity achieved by liquid resonators is several orders higher than that of solid resonators. To increase the stability of the droplet, light in the resonator could be coupled using a tapered fiber instead of free-space coupling [42]. Further research will be conducted to create a humidity sensor that could be used for industrial needs.

## Figures and Tables

**Table 1:** Overview of previous research on WGM humidity sensors.

Sensing Material/Type	Sensitivity	*Q* Factor, Wavelength, Type of Measurement	Precision and Resolution, Detection Limit	Ref.
Silica microbottle (Figure 1a)	0.049 dB/% RH	*Q* = 10^4^, excitation by tunable laser (1551–1559 nm), transmission spectrum	-	[18]
Silica microsphere coated with a layer of Agarose hydrogel (Figure 1b)	518 pm/% RH (30–70% RH)	*Q* = 10^4^, excitation by a broadband superluminescent light source, spectrum analysed with OSA	2.0227 pm (resolution) 1.15 × 10^−1^% RH (det. limit)	[19]
PMMA microspheres with different diameters (Figure 1c)	47 pm/% RH	*Q*–N/A, excitation by tunable laser (centered at 635.5 nm), mode map of many spheres (intensity pattern)	-	[20]
Glycerol microsphere doped with rhodamine 6G dye (Figure 1d)	2 nm/% RH (45–65% RH)	*Q* = 10^3^, excitation 532 nm, rhodamine 6G spectrum (550–650 nm)	3 × 10^−3^% RH (detection limit from COMSOL simulation proposed prototype)	[15]
Roll up polymer/oxide/polymer nanomembranes	130 pm/% RH (5–97% RH)	Excitation 514.5 nm, photoluminescence spectrum (650–675 nm)	-	[21]
SU-8 polymer microdisk	78.4 pm/% RH (0–5% RH); 23.5 pm/% RH (45–50% RH)	*Q* = 10^3^, excitation by tunable laser (1500–1620 nm), transmission spectrum	0.03% RH (detection limit)	[22]
Sol-gel-based integrated microring	16 pm/% RH	*Q* = 10^4^, excitation by tunable laser (2 nm range), sol-gel clad spectrum (1310–1311 nm)	0.16% RH (detection limit)	[23]
Silica microtoroid coated with nm-scale polymer (pNIPAAm)	12.98 pm/% RH	*Q* = 10^5^, excitation by tunable laser (centered at 980 nm), transmission spectrum	-	[24]
Silica microsphere coated with a thin layer of Agarose hydrogel (Figure 1b)	0.71 pm/% RH (1–25% RH)	*Q* = 10^6^, excitation by tunable laser (1490–1640 nm), transmission spectrum (oscilloscope)	43.58 fm (resolution) 6.13 × 10^−2^% RH (detection limit)	[9]
Silica microsphere (WGM excited using a tapered fiber)	0.11 dB/% RH (20–70% RH); 0.21 dB/% RH (70–90% RH)	*Q* = 10^4^, broadband source (centered at 1550 nm), transmission spectrum (OSA)	0.02 nm and 0.001 dB (resolution)	[25]

## Data Availability

Data associated with this paper are available upon request.

## Supplementary Materials

The following are available online at https://www.mdpi.com/1424-8220/21/5/1746/s1, Figure S1: Transmission spectra of glycerol, water and 50:50 glycerol-water mix, Video S1: Resonant modes wavelength shift due to humidity change.
