# Supplementary material for: High-Sensitivity Whispering Gallery Mode Humidity Sensor Based on Glycerol Microdroplet Volumetric Expansion
Source: Sensors (Basel). 2021 Mar 3;21(5):1746. doi: 10.3390/s21051746 (PMC7959475; doi:10.3390/s21051746)
Supplement: Supplementary file 1 [file sensors-21-01746-s001.zip › sensors-1110558-supplementary-final.docx]

*Supplementary Materials*

High-Sensitivity Whispering Gallery Mode Humidity Sensor Based on Glycerol Microdroplet Volumetric Expansion

Pauls Kristaps Reinis *, Lase Milgrave *, Kristians Draguns, Inga Brice, Janis Alnis and Aigars Atvars

Quantum optics laboratory, Institute of Atomic Physics and Spectroscopy, University of Latvia, LV-1586 Riga, Latvia; kristians.draguns@lu.lv (K.D.); inga.brice@lu.lv (I.B.); janis.alnis@lu.lv (J.A.); aigars.atvars@lu.lv (A.A.)

* Correspondence: pauls_kristaps.reinis@lu.lv (P.K.R.); lase.milgrave@lu.lv (L.M.)


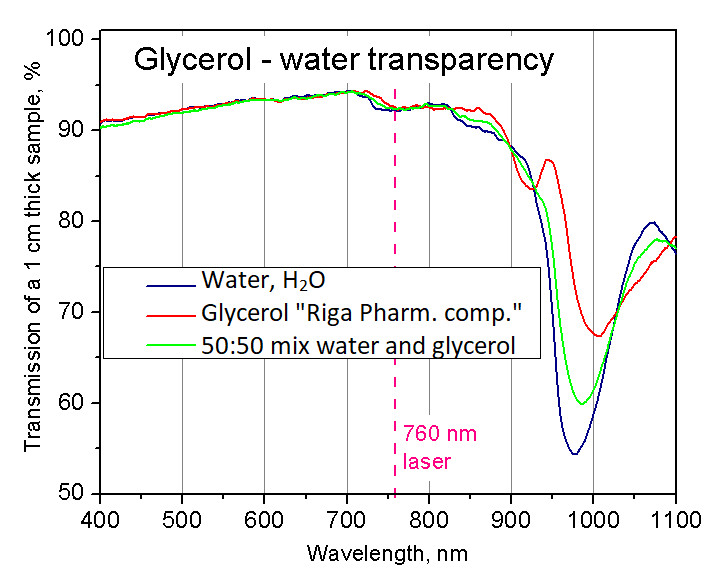


**Figure S1.** Experimentally measured transmission spectra of glycerol, water, and 50:50 glycerol–water mix. We measured glycerol and water absorption with a grating spectrometer using a 1-cm-thick cuvette. Measurements revealed that the wavelength region around 700 nm had low absorption, and that one should not use wavelengths around 980 nm because of strong water absorption. Thus, a 760 nm laser was chosen for our experiments to excite whispering gallery modes in the glycerol microdroplet.
